# MGFR-ViT: A Multi-Scale Gated Feature Refinement Vision Transformer for Vibration-Based Fault Diagnosis

**DOI:** 10.3390/s26144652

**Published:** 2026-07-22

**Authors:** Yan Yan, Ting Shang, Kun Jia, Songnan Yang, Haiyan Cheng, Yuxing Li, Wei Quan

**Affiliations:** 1 School of Automation and Information Engineering, Xi’an University of Technology, Xi’an 710048, China; st107@xaut.edu.cn (T.S.); yang.son.nan@xaut.edu.cn (S.Y.); chenghaiyan@xaut.edu.cn (H.C.); liyuxing@xaut.edu.cn (Y.L.); wei.quan@xaut.edu.cn (W.Q.); 2 School of Mechanical Engineering, Xi’an University of Technology, Xi’an 710048, China; 1230211020@stu.xaut.edu.cn

**Keywords:** fault diagnosis, vision transformer, multi-scale feature refinement, local enhancement mechanism

## Abstract

To address the limited extraction of local fault features, ineffective fusion of multi-scale fault information and interference from redundant noise in vibration-based fault diagnosis of rolling bearings and gears under complex operating conditions, a multi-scale gated feature refinement Vision Transformer (MGFR-ViT), is proposed. First, one-dimensional vibration signals are reconstructed as two-dimensional vibration matrices, making them compatible with patch embedding and Vision Transformer-based feature modeling. A locally enhanced Vision Transformer module is then developed by incorporating a local enhancement mechanism into the standard Vision Transformer architecture, thereby improving the extraction of locally fault-sensitive features while preserving global dependency modeling. Furthermore, a multi-scale gated feature refinement module is introduced to adaptively enhance fault-relevant information and suppress redundant features and noise through parallel multi-scale convolutions with different receptive fields, channel interaction, and gated weighting. Finally, global average pooling and a fully connected classifier are employed for fault classification. Experiments conducted on bearing and gear datasets demonstrated that MGFR-ViT achieved superior diagnostic performance and feature separability compared with several representative fault diagnosis models. Ablation studies further validated the effectiveness and complementarity of the proposed modules. These results indicate that MGFR-ViT provides an effective feature-learning framework for vibration-based fault diagnosis of rotating machinery.

## 1. Introduction

Rotating machinery is widely used in critical sectors, including industrial manufacturing, energy systems, rail transportation, and aerospace. Its operating condition directly affects equipment safety, production continuity, and system reliability. As core transmission components of rotating machinery, bearings and gears are continuously exposed to alternating loads, lubricant degradation, impact-induced wear, and environmental disturbances during long-term operation, which may result in localized damage or performance deterioration. Failure to detect incipient faults in a timely manner may lead to unplanned downtime, increased maintenance costs, and even severe safety incidents. Therefore, the development of accurate, robust, and adaptable fault diagnosis methods for rotating machinery is of considerable engineering significance and practical value [1,2,3].

Vibration signals directly reflect the dynamic responses of mechanical equipment during operation and are among the most commonly used monitoring signals for rotating machinery fault diagnosis. Conventional fault diagnosis methods generally rely on handcrafted features, such as time-domain statistical indicators, frequency-domain characteristics, and time–frequency representations. These features are typically combined with machine learning classifiers, including support vector machines [4], random forests [5], and K-nearest neighbors [6], to identify different fault states. Although these methods provide a certain degree of interpretability and have been widely applied in early studies of mechanical fault diagnosis, their performance depends heavily on expert knowledge and the quality of handcrafted features. Under complex operating conditions characterized by similar fault patterns, substantial noise interference, or weak fault signatures, manually designed features often fail to adequately capture subtle differences among health states, thereby limiting model generalization and practical applicability.

In recent years, deep learning methods have been extensively applied to mechanical fault diagnosis because of their powerful automatic feature-learning capabilities. Convolutional neural networks can automatically extract local patterns from raw signals or two-dimensional representations, substantially reducing the dependence on handcrafted features [7,8]. However, convolutional operations are inherently constrained by local receptive fields, and deep network architectures are generally required to capture long-range dependencies, which increases model complexity. Recurrent neural networks and their variants can characterize the temporal dynamics of sequential data but remain limited in long-sequence modeling and parallel computational efficiency. In contrast, Transformers and their variants use self-attention mechanisms to directly capture global dependencies among different sequence positions. They therefore offer distinct advantages in learning complex feature representations and provide a promising framework for vibration-based fault diagnosis [9,10,11,12].

As a representative extension of the Transformer architecture to visual tasks, the Vision Transformer (ViT) partitions an input image or two-dimensional matrix into a sequence of patches and uses multi-head self-attention to model global relationships among them, thereby providing a strong capability for representing long-range dependencies [13,14]. For vibration-based fault diagnosis, one-dimensional vibration signals can be reconstructed into two-dimensional matrices, allowing ViT to capture structural correlations among different regions of the reconstructed signals. However, the standard ViT relies primarily on global self-attention and therefore has a limited ability to capture local impacts and transient variations. Moreover, vibration responses induced by different fault types and damage severities exhibit distinct scale-dependent characteristics. A single receptive field is thus insufficient to simultaneously characterize fine local details and broader structural variations. In addition, multi-scale feature fusion may introduce channel isolation, redundant information, and noise interference, thereby reducing the discriminability of the learned fault representations.

To address these limitations, a multi-scale gated feature refinement Vision Transformer (MGFR-ViT) is proposed for vibration-based fault diagnosis. First, one-dimensional vibration signals are reconstructed into two-dimensional vibration matrices and transformed into feature sequences through patch embedding, enabling global relationships among different vibration regions to be modeled. Second, a locally enhanced Vision Transformer (LE-ViT) is developed by embedding a local enhancement module (LEM) into the feed-forward network of the standard ViT. This design preserves the global dependency modeling capability of ViT while improving its sensitivity to local impacts and transient variations. Furthermore, a multi-scale gated feature refinement module (MGFR) is introduced. Small-scale, large-scale, and dilated grouped convolutions are employed to extract fault features under different receptive fields. Channel interaction, gated weighting, and residual connections are then integrated to fuse multi-scale information, enhance fault-sensitive features, and suppress redundant information and noise. Finally, global average pooling and a fully connected classifier are used to perform fault classification.

The main contributions of this paper are summarized as follows:

1. An MGFR-ViT model is proposed for vibration-based fault diagnosis. Feature learning is performed through a progressive process of local impact compensation and multi-scale discriminative feature refinement, enabling periodic correlations, transient impacts, and scale-dependent variations to be jointly characterized while suppressing fault-irrelevant responses.
2. An LE-ViT module is developed by embedding the LEM into the feed-forward network of ViT. After global contextual relationships are established through multi-head self-attention, local neighborhood compensation is performed. Consequently, the ability to represent local impacts, transient variations, and fine-grained fluctuations is improved without compromising global dependency modeling.
3. An MGFR module is proposed to model short-duration impacts, intermediate-scale modulations, and broad structural responses in parallel through grouped convolutions with different effective receptive fields. Cross-group information interaction is restored through channel shuffling. A feature-level gating matrix is then generated while preserving the spatial structure, and residual connections are incorporated to enhance fault-related features, suppress noise, and retain the original information.

The remainder of this paper is organized as follows. Section 2 reviews the research related to this study. Section 3 describes the architecture of the proposed MGFR-ViT in detail. Section 4 presents the datasets, experimental settings, comparative results, and ablation studies. Finally, Section 5 summarizes the main findings and discusses future research directions.

## 2. Related Work

### 2.1. Vision Transformer-Based Fault Diagnosis Methods

ViT was originally developed for image recognition. Its core idea is to divide an input image into a sequence of patches and model the global dependencies among them through multi-head self-attention [15]. By overcoming the limitations imposed by local receptive fields, ViT exhibits distinct advantages in global feature representation and long-range dependency modeling.

In recent years, ViT has been increasingly applied to fault diagnosis. For vibration signals, one-dimensional time series can be converted into two-dimensional representations through matrix reconstruction or related transformation methods. Such two-dimensional vibration representations not only preserve the temporal structure of the original signals but also spatially characterize the latent relationships among different temporal segments or frequency components. Based on this property, ViT can exploit self-attention to capture global relationships among different regions of a two-dimensional vibration representation, thereby improving the recognition of complex fault patterns. Existing studies have shown that transforming vibration signals into two-dimensional images or matrix representations and subsequently modeling them with ViT can achieve favorable classification performance in fault diagnosis tasks involving bearings, gears, and other rotating machinery [16,17,18].

However, fault diagnosis based solely on ViT still has several limitations. On the one hand, mechanical vibration signals commonly contain local impacts, transient variations, and periodic weak impulses, whereas the standard ViT mainly relies on global self-attention and lacks the local inductive bias inherent in convolutional neural networks [19]. On the other hand, two-dimensional vibration representations generally contain background noise and redundant information. Directly using ViT-derived features for classification may therefore be insufficient to emphasize critical fault signatures while suppressing irrelevant interference [20,21].

In summary, ViT provides an effective framework for global feature modeling in vibration-based fault diagnosis. Nevertheless, its capabilities for extracting locally fault-sensitive features and refining discriminative representations remain limited. Therefore, incorporating local enhancement mechanisms and multi-scale feature refinement strategies while preserving the global dependency modeling capability of ViT represents an effective approach to improving fault diagnosis performance.

### 2.2. Multi-Scale Gated Feature Refinement Methods

Multi-scale feature modeling is a commonly used feature enhancement strategy in mechanical fault diagnosis. Its core principle is to capture fault-related information at multiple scales through feature extraction branches with different receptive fields. Mechanical vibration signals generally exhibit pronounced multi-scale characteristics. Local defects are often manifested as short-duration impacts, transient variations, or high-frequency fluctuations, whereas periodic modulation and changes in operating conditions may appear as dynamic variations over longer time scales. Multi-scale modeling can therefore characterize both local details and broader structural patterns, thereby improving the representation of complex fault modes [22].

In recent years, multi-scale convolutions, attention mechanisms, and gating mechanisms have been increasingly introduced into rotating machinery fault diagnosis. Multi-scale convolutional methods generally employ convolution kernels of different sizes, dilated convolutions, or parallel convolutional branches to extract fault features under different receptive fields, thereby enhancing sensitivity to local impacts and periodic fault patterns. Attention and gating mechanisms can adaptively generate weight distributions according to the input features, enabling fault-sensitive regions or critical channels to be emphasized while redundant information and noise are suppressed [23,24]. In addition, grouped convolutions, pointwise convolutions, and channel shuffling have been employed to reduce computational cost and strengthen inter-channel information interaction [25,26]. Residual connections have also been adopted to preserve useful original information and improve training stability [27].

However, existing feature refinement methods still exhibit several limitations. On the one hand, some methods fuse multi-scale features through direct concatenation or element-wise addition without adaptively evaluating the importance of individual scales, which may introduce redundant information and noise. On the other hand, multi-scale convolutions and attention mechanisms are often used as independent feature extraction modules, making it difficult to fully exploit the complementary relationship between global dependency modeling and multi-scale local feature enhancement.

In summary, further investigation is required to achieve effective multi-scale fault information fusion, channel interaction, and redundant feature suppression based on the global representations learned by ViT.

## 3. Methodology

An MGFR-ViT model is proposed for vibration-based fault diagnosis. First, one-dimensional vibration signals are reconstructed into two-dimensional vibration matrices to make them compatible with the input format of ViT. The LE-ViT module is then employed to extract features that capture both global dependencies and locally fault-sensitive information. Subsequently, the MGFR module adaptively selects and enhances fault features at different scales. Finally, global average pooling and a fully connected classifier are used to identify the fault states.

### 3.1. Overall Framework of MGFR-ViT

**Figure 1:**
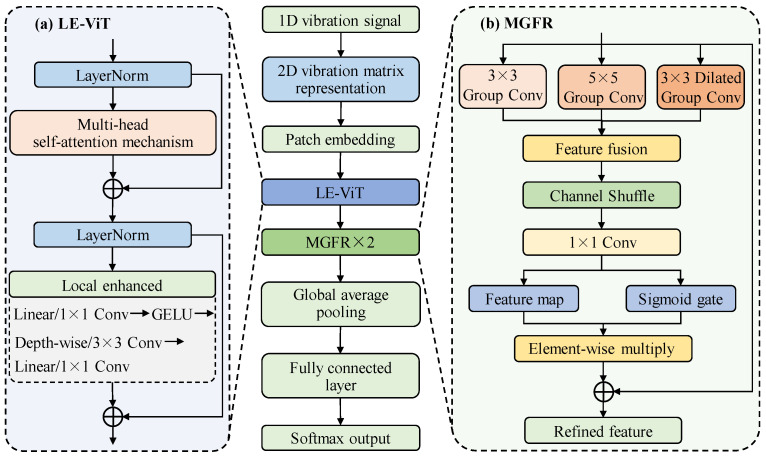
Overall Architecture of the Proposed MGFR-Vit Model.

The proposed MGFR-ViT consists of three main components: the LE-ViT feature modeling module, the MGFR feature refinement module, and the fault classification module. The overall architecture is illustrated in Figure 1.

First, each one-dimensional vibration signal is segmented using a fixed window length and reconstructed into a two-dimensional vibration matrix to match the input format of ViT. The matrix is then divided into a sequence of patches, which are linearly projected into embedded representations. Positional encodings are subsequently added to preserve the spatial relationships among the patches.

During feature modeling, the LE-ViT module is employed to capture global dependencies within the embedded sequence. An LEM is incorporated into the standard ViT architecture to introduce local inductive bias through depthwise convolution. This design enables the model to capture long-range dependencies among different regions while enhancing its sensitivity to local impacts and transient fault signatures.

The features produced by LE-ViT are then forwarded to the MGFR module for further refinement. Parallel multi-scale branches are constructed using 3 × 3 grouped convolution, 5 × 5 grouped convolution, and 3 × 3 dilated grouped convolution to extract fault features under different receptive fields. Feature fusion, channel shuffling, 1 × 1 convolution, and a Sigmoid-based gating mechanism are subsequently applied to facilitate inter-channel interaction and adaptive feature selection. Finally, a residual connection is introduced to preserve useful original information and generate enhanced discriminative fault representations.

The refined features are then reduced through global average pooling and fed into a fully connected layer followed by a Softmax classifier to determine the final fault category. Through this architecture, MGFR-ViT jointly integrates global dependency modeling, local feature enhancement, and multi-scale feature refinement, thereby improving the recognition of complex vibration fault patterns.

### 3.2. Local-Enhanced Vision Transformer

ViT employs multi-head self-attention mechanism to establish global dependencies among different patches, enabling correlations between adjacent and distant regions in a two-dimensional vibration matrix to be effectively captured. However, the feed-forward network in the standard ViT primarily performs an independent channel-wise transformation for each patch and lacks explicit information interaction among neighboring patches. When a local fault response is concentrated within only a few adjacent regions, its fine-grained structural information may be diluted by the globally aggregated features.

To address this limitation, an LE-ViT module is designed, as illustrated in Figure 1a. The standard layer normalization, multi-head self-attention mechanism, and residual connection structures of ViT are retained, while an LEM is embedded within the feed-forward network to locally enhance the attention-derived features. It should be emphasized that the LEM is not implemented as an independent convolutional module placed before or after ViT. Instead, it is integrated into the feed-forward transformation, allowing the global contextual features learned by multi-head self-attention mechanism to be further calibrated through local neighborhood interactions.

For an input feature $Z_{l-1}$, the computation of the *l*-th LE-ViT module is expressed as

(1) $$Z_{l}^{\prime }=Z_{l-1}+\mathrm{MSA}(\mathrm{LN}(Z_{l-1}))$$

(2) $$Z_{l}=Z_{l}^{\prime }+\mathrm{LEM}(\mathrm{LN}(Z_{l}^{\prime }))$$

where MSA(⋅), LEM(⋅), and LN(⋅) denote the multi-head self-attention mechanism, local enhancement operation, and layer normalization, respectively.

In the multi-head self-attention mechanism, the input feature is projected into the query, key, and value representations as

(3) $$Q={ZW}_{Q},K={ZW}_{K},V={ZW}_{V}$$

where $W_{Q},W_{K},W_{V}$ are learnable parameters. The self-attention operation is calculated as

(4) $$\mathrm{Attention}(Q,K,V)=\mathrm{Softmax}(\frac{QK^{T}}{\sqrt{d}})V$$

where *d* denotes the feature dimension. Multi-head self-attention models dependencies in different subspaces through multiple attention heads in parallel, thereby improving the global feature representation capability.

The proposed LEM introduces depthwise convolution into the channel transformation process and is formulated as

(5) $$\mathrm{LEFFN}(Z)={\mathrm{Conv}}_{1\times 1}({\mathrm{DWConv}}_{3\times 3}(\mathrm{GELU}({\mathrm{Conv}}_{1\times 1}(Z))))$$

where ${\mathrm{Conv}}_{1\times 1}(\cdot )$ performs channel-wise projection, ${\mathrm{DWConv}}_{3\times 3}(\cdot )$ denotes a 3 × 3 depthwise convolution, and GELU(⋅) is the nonlinear activation function. The 1 × 1 convolutions adjust the number of feature channels and strengthen channel-wise representation, whereas the 3 × 3 depthwise convolution facilitates spatial information interaction within local neighborhoods. Consequently, the sensitivity of the model to local impacts and fine-grained fault patterns is improved.

The key distinction between LE-ViT and the standard ViT lies in the feed-forward transformation. The standard ViT independently transforms the channel representation of each patch, whereas LE-ViT explicitly introduces local neighborhood interactions into this process. Accordingly, multi-head self-attention mechanism establishes global associations across different regions, while LEM performs local compensation on the globally contextualized features. A progressive feature-processing procedure of global dependency modeling followed by local response calibration is therefore formed. Without altering the overall ViT encoder architecture, this design improves the representation of local fault responses while avoiding the substantial parameter increase associated with additional independent convolutional branches.

### 3.3. Multi-Scale Gated Feature Refinement Module

Although LE-ViT can simultaneously extract global dependencies and local fault-sensitive features, its output features may still contain redundant information and noise interference. Mechanical vibration signals usually exhibit distinct multi-scale characteristics. Local damage is often manifested as short-term impacts, transient mutations, or high-frequency fluctuations, whereas periodic faults and operating condition variations may lead to dynamic changes over a wider temporal range. Therefore, single-scale feature extraction is insufficient to fully characterize these complex fault patterns.

To further improve feature discriminability, an MGFR module was designed, as shown in Figure 1b. In this module, parallel multi-scale grouped convolutions are used to extract feature information under different receptive fields. Feature fusion, channel shuffle, 1 × 1 convolution, a sigmoid gating mechanism, and residual connections are then combined to adaptively enhance fault-sensitive features while suppressing redundant information.

Given an input feature $F_{in}\in {\mathbb{R}}^{C\times H^{\prime }\times W^{\prime }}$, where *C* denotes the number of channels, and *H*′ and *W*′ denote the spatial dimensions of the feature map. MGFR first adopts three parallel branches to extract features at different scales:

(6) $$F_{1}={\mathrm{GConv}}_{3\times 3}(F_{in})$$

(7) $$F_{2}={\mathrm{GConv}}_{5\times 5}(F_{in})$$

(8) $$F_{3}={\mathrm{DConv}}_{3\times 3}(F_{in})$$

where ${\mathrm{GConv}}_{3\times 3}(\cdot )$ and ${\mathrm{GConv}}_{5\times 5}(\cdot )$ denote 3 × 3 and 5 × 5 grouped convolutions, respectively, and ${\mathrm{DConv}}_{3\times 3}(\cdot )$ denotes a 3 × 3 dilated grouped convolution. The small-scale convolution is mainly used to capture local impacts and detailed features, whereas the large-scale convolution and dilated convolution are used to enlarge the receptive field and extract fault structural information over a wider range.

The three branch features are then fused by concatenation:

(9) $$F_{ms}=\mathrm{Concat}(F_{1},F_{2},F_{3})$$

where Concat(⋅) denotes the feature concatenation operation.

Since grouped convolution may weaken information interaction among different channel groups while reducing the number of parameters, a channel shuffle operation is further introduced to promote cross-group feature interaction:

(10) $$F_{f}={\mathrm{Conv}}_{1\times 1}(\mathrm{Shuffle}(F_{ms}))$$

where Shuffle(⋅) denotes the channel shuffling operation.

After multi-scale feature fusion, a gating mechanism is introduced to adaptively select fault-sensitive features. The gating weight is calculated as

(11) $$G=\sigma ({\mathrm{Conv}}_{3\times 3}(F_{f}))$$

where σ denotes the Sigmoid activation function, and **G** denotes the gating weight. The fused feature is then weighted by the gating mechanism:

(12) $$F_{g}=F_{f}⊙G$$

where ⊙ denotes element-wise multiplication. This process enhances the responses of key features related to fault categories while suppressing noise and redundant information.

Finally, to avoid the loss of original effective information during feature refinement, a residual connection is introduced:

(13) $$F_{out}=F_{in}+F_{g}$$

Through the residual connection, the model can retain the original feature information while improving training stability and alleviating information attenuation during deep feature transformation.

In the proposed model, MGFR is stacked sequentially to further strengthen the representation capability of multi-scale fault features. Through the joint effects of multi-scale convolution, channel interaction, gating-based weighting, and residual refinement, MGFR effectively improves feature discriminability and enables the model to focus more on fault-related key information.

### 3.4. Fault Classification Module

After being processed by LE-ViT and MGFR, refined features containing global dependencies, local sensitive features, and multi-scale discriminative information are obtained. To reduce the number of model parameters and alleviate the risk of overfitting, global average pooling is adopted instead of the traditional flatten operation to compress the spatial dimensions. For the output feature $F_{out}\in {\mathbb{R}}^{C\times H^{\prime }\times W^{\prime }}$, global average pooling is defined as

(14) $$f_{c}=\frac{1}{H^{\prime }W^{\prime }}\sum_{i=1}^{H^{\prime }}\sum_{j=1}^{W^{\prime }}F_{out}(c,i,j)$$

where $f_{c}$ denotes the global average response of the *c-th* channel. After global average pooling, a one-dimensional feature vector is obtained as

(15) $$f=[f_{1},f_{2},\cdots ,f_{C}]$$

Subsequently, the feature vector is fed into a fully connected layer, and the predicted probability of each fault category is obtained through the Softmax function:

(16) $$\hat{y}=\mathrm{Softmax}(Wf+b)$$

where **W** and **b** denote the weight matrix and bias term of the fully connected layer, respectively, and $\hat{y}$ denotes the predicted class probability.

During model training, the cross-entropy loss function is employed:

(17) $$L=-\frac{1}{M}\sum_{i=1}^{M}\sum_{k=1}^{K}y_{i,k}\log({\hat{y}}_{i,k})$$

where *M* denotes the number of samples, *K* denotes the number of fault categories, $y_{i,k}$ denotes the true label of the *i*-th sample for the *k*-th class, and ${\hat{y}}_{i,k}$ denotes the corresponding predicted probability.

Through global average pooling, the fully connected layer, and the Softmax classifier, MGFR-ViT maps the refined high-dimensional fault features into the final category outputs, thereby achieving end-to-end vibration-based fault diagnosis.

## 4. Experiments and Results

All experiments were conducted on a computing platform equipped with an NVIDIA RTX 3060 GPU (Nvidia, Santa Clara, CA, USA), an Intel Core i9-12900K CPU, and 64 GB of RAM (Intel, Santa Clara, CA, USA). Model training and testing were performed using MATLAB R2024b.

### 4.1. Dataset and Experimental Setup

#### 4.1.1. Paderborn University Bearing Dataset

The rolling bearing dataset provided by Paderborn University (PU) [28] contains high-resolution vibration signals collected from three bearing categories: six healthy bearings, 12 bearings with artificially induced damage, and 14 bearings with real damage obtained through accelerated lifetime tests. As shown in Table 1, four bearings were selected from each category to ensure sufficient sample availability and class balance, resulting in 12 condition classes covering healthy, inner-race fault, and outer-race fault states. The vibration signals were sampled at 64 kHz, and the detailed class definitions are presented in Table 1. A total of 1200 samples were extracted, with 100 samples assigned to each class, and were randomly divided into training and test sets at a ratio of 4:1.

#### 4.1.2. University of Connecticut Gear Dataset

The gear dataset provided by the University of Connecticut (UC) [29] was collected from a two-stage gearbox with replaceable gears. The vibration signals were acquired at a sampling frequency of 20 kHz. As shown in Table 2, this dataset contains nine typical gear health conditions, including healthy condition, missing tooth, root crack, spalling, and five levels of tooth tip breakage, where the tooth tip breakage was gradually divided according to the physical severity of the fault. For each fault category, 104 samples were generated in this study, resulting in a total of 936 samples. This setting ensures sample balance among different categories. Subsequently, all samples were randomly divided at a ratio of 10:3 to construct the training and testing sets for model training and performance evaluation.

#### 4.1.3. Case Western Reserve University Bearing Dataset

The Case Western Reserve University (CWRU) bearing dataset is a widely used public benchmark for rotating machinery fault diagnosis [30]. In this study, drive-end vibration signals sampled at 48 kHz were selected. Ten health conditions were considered, including one normal condition and nine fault conditions involving rolling-element, inner-race, and outer-race faults. Each fault type included three defect sizes: 0.007, 0.014, and 0.021 in. A total of 1000 samples were extracted, with 100 samples assigned to each class. The samples were divided into training and test sets at a ratio of 7:3. The detailed class definitions are presented in Table 3.

Model performance was evaluated using four metrics: accuracy, precision, recall, and F1-score. The corresponding calculation formulas are given in Equations (18)–(21):

(18) $$Accuracy=\frac{TP+TN}{TP+TN+FP+FN}$$

(19) $$Precision=\frac{TP}{TP+FP}$$

(20) $$Recall=\frac{TP}{TP+FN}$$

(21) $$F1\_Score=\frac{2\times Precision\times Recall}{Precision+Recall}$$

where *TP* denotes true positives, *TN* denotes true negatives, *FP* denotes false positives, and *FN* denotes false negatives.

### 4.2. Discussion

**Figure 2:**
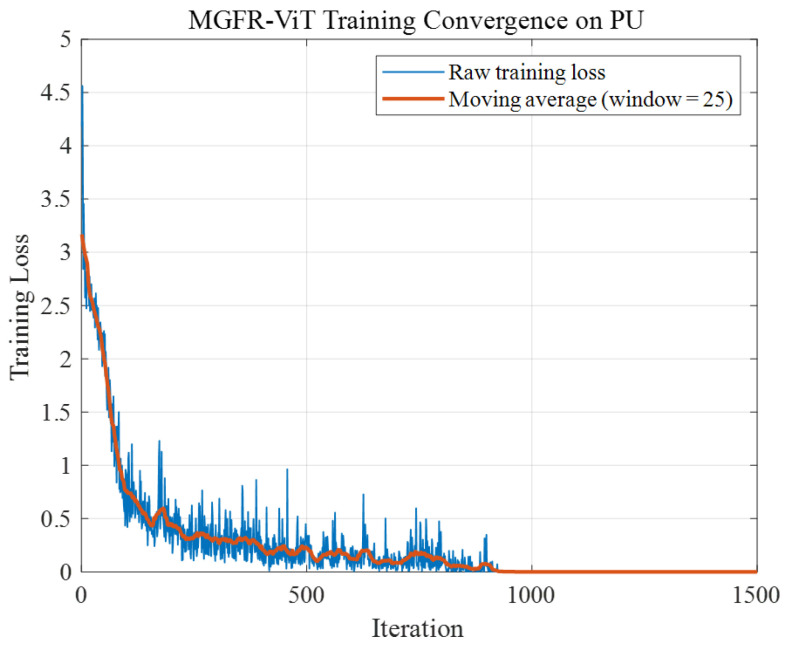
Training Loss Convergence Curve of MGFR-Vit on the PU Dataset.

As shown in Figure 2, the training loss of MGFR-ViT on the PU dataset decreased rapidly during the initial stage, indicating that the model quickly learned the principal fault-related characteristics of the bearing vibration signals. As the number of iterations increased, the rate of loss reduction gradually slowed, and the loss stabilized at a low level after approximately 900 iterations. No evident oscillation or divergence was observed during the later stage of training, demonstrating a stable convergence process on the PU dataset.

**Figure 3:**
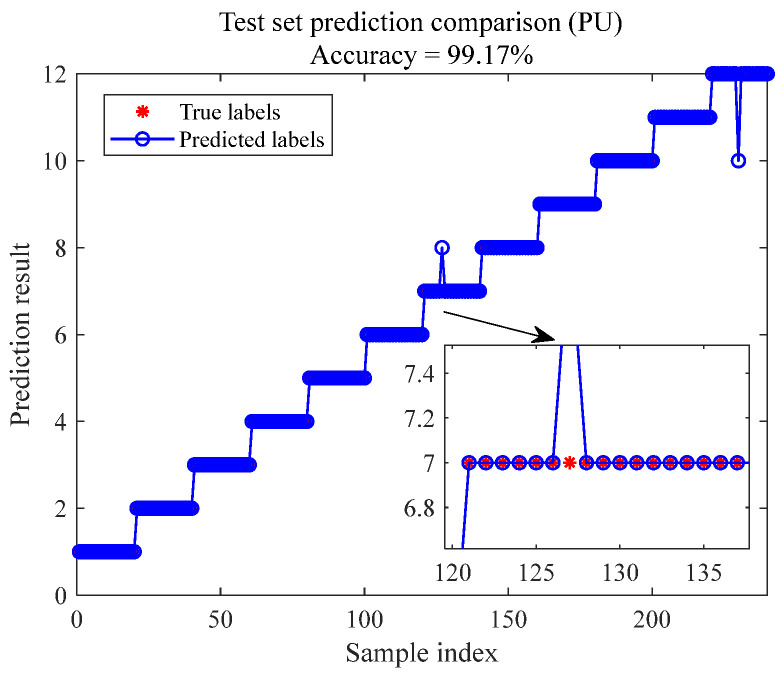
Comparison of True and Predicted Labels on the PU Dataset.

Figure 3 compares the true and predicted labels of the test samples from the PU dataset. Overall, the predicted labels, represented by blue circles, almost completely overlapped with the true labels, represented by red asterisks. This result indicates that the proposed model effectively captured the discriminative features of different fault classes and accurately identified all 12 operating conditions. Only a few minor prediction errors were observed, without affecting the overall classification trend, demonstrating the strong stability and robustness of the model.

**Figure 4:**
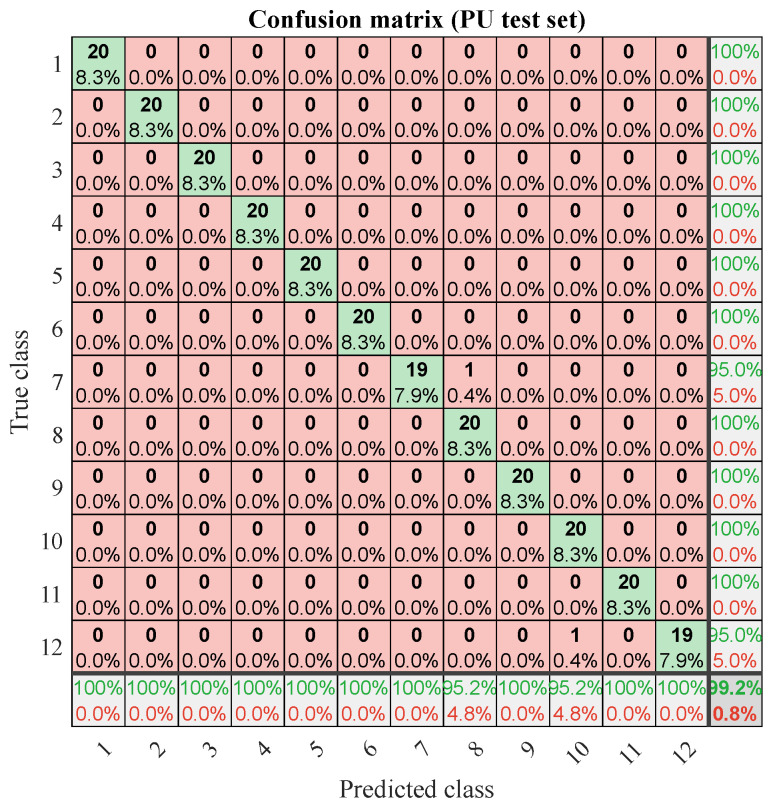
Confusion Matrix for the PU Dataset.

Figure 4 presents the corresponding confusion matrix. Most classes achieved a classification accuracy of 100%, as indicated by the dominant values along the main diagonal, demonstrating the strong discriminative capability of the model for different fault characteristics. Only a few misclassifications occurred in Classes 7 and 12. Specifically, one sample from Class 7 was misclassified as an adjacent class, and one sample from Class 12 was also incorrectly classified, resulting in a class-specific accuracy of approximately 95% for each class. Overall, the prediction accuracy reached 99.2%, with a misclassification rate of only 0.8%, further confirming the high diagnostic accuracy of the model. The training time of MGFR-ViT on the PU dataset was 61.685 s, and the average inference time per sample was 7.712 ms. Sequential inference for all 240 test samples required approximately 1.851 s.

Overall, the proposed method achieved excellent classification performance on the PU dataset, with high overall accuracy and minimal interclass confusion. These results demonstrate that MGFR-ViT effectively extracted discriminative information from the bearing vibration signals and exhibited favorable generalization capability and potential for engineering applications.

**Figure 5:**
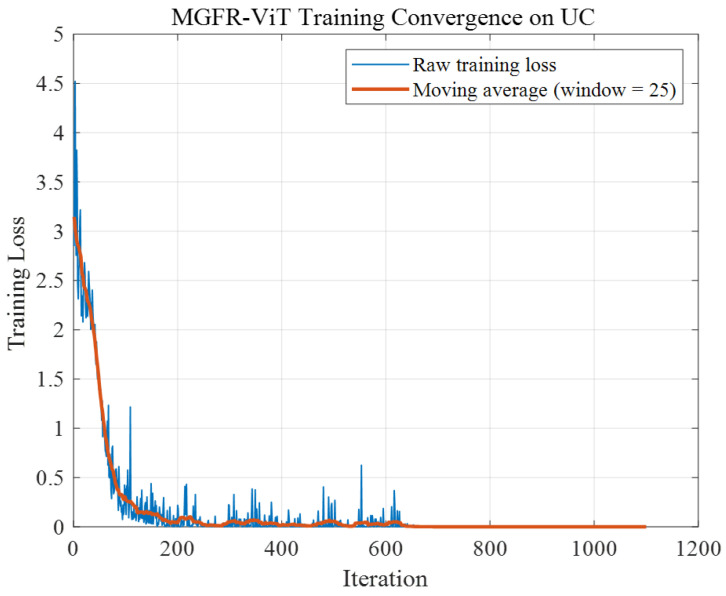
Training Loss Convergence Curve of MGFR-Vit on the UC Dataset.

As shown in Figure 5, the training loss of MGFR-ViT on the UC dataset decreased rapidly during the initial stage and became essentially stable after approximately 600 iterations. Compared with the PU dataset, the loss on the UC dataset declined more quickly and remained close to zero during the later stage of training. Although the raw training loss exhibited several transient fluctuations, the moving-average curve decreased smoothly, with no sustained increase or divergence. These results demonstrate that the model achieved stable optimization and favorable convergence in the UC gear fault diagnosis task.

**Figure 6:**
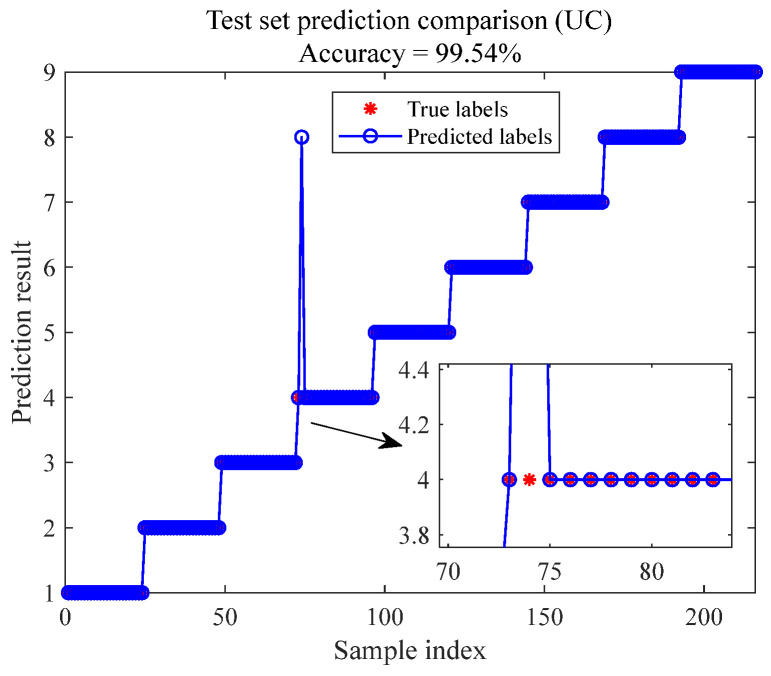
Comparison of True and Predicted Labels on the UC Dataset.

Figure 6 compares the true and predicted labels of the test samples from the UC dataset. Overall, the predicted labels, represented by blue circles, closely matched the true labels, represented by red asterisks. Most samples were classified correctly, with only minor deviations at a few positions. For example, slight prediction fluctuations occurred near Class 4, although the predicted labels still closely followed the overall variation in the true classes. These results indicate that the proposed model effectively learned discriminative representations of different gear fault patterns and exhibited strong stability and generalization in the multiclass classification task.

**Figure 7:**
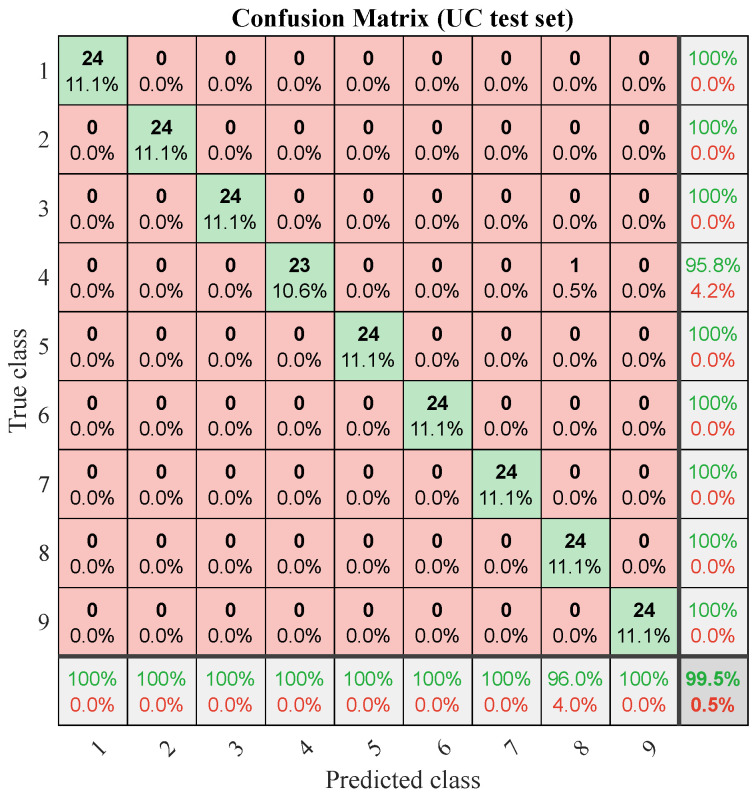
Confusion Matrix for the UC Dataset.

Figure 7 presents the corresponding confusion matrix. Most samples are concentrated along the main diagonal, and most classes achieved a classification accuracy of 100%, indicating that the model correctly identified nearly all fault conditions. Only Class 4 exhibited a minor misclassification, with one sample incorrectly assigned to another class, resulting in a class-specific accuracy of 95.8%. Overall, the classification accuracy reached 99.5%, whereas the misclassification rate was only 0.5%, demonstrating the high diagnostic accuracy of MGFR-ViT on the UC dataset. The training time was 38.104 s, and the average inference time per sample was 8.220 ms. Sequential inference for all 216 test samples required approximately 1.776 s.

Overall, the proposed method achieved excellent classification performance on the UC gear dataset. The predicted labels closely matched the true labels, and minimal interclass confusion was observed in the confusion matrix, confirming the effectiveness and reliability of the model for complex gear fault diagnosis. These findings further demonstrate that MGFR-ViT effectively extracts fault-discriminative features and accurately distinguishes gear faults with different types and damage severities, indicating strong potential for engineering applications.

**Figure 8:**
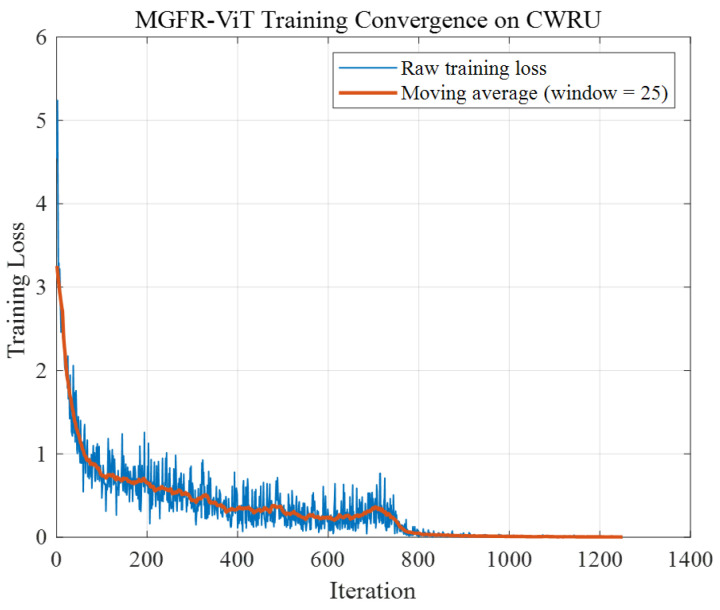
Training Loss Convergence Curve of MGFR-Vit on the CWRU Dataset.

As shown in Figure 8, the training loss of MGFR-ViT on the CWRU dataset decreased rapidly during the initial stage, indicating that the model quickly learned the key fault-related characteristics of the bearing vibration signals. As the number of iterations increased, the loss continued to decrease and gradually stabilized at a value close to zero after approximately 800 iterations. Although the raw loss curve exhibited several fluctuations because of mini-batch stochastic optimization, the moving-average curve maintained a smooth downward trend. No evident oscillation or divergence was observed during the later training stage, demonstrating favorable convergence and training stability on the CWRU bearing fault diagnosis task.

**Figure 9:**
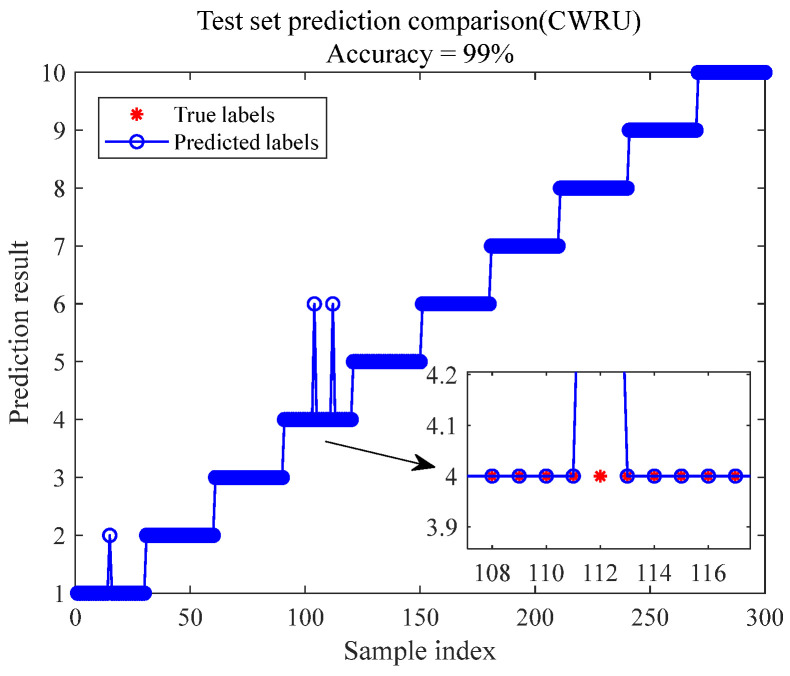
Comparison of True and Predicted Labels on the CWRU Dataset.

Figure 9 presents the test-set prediction results of MGFR-ViT on the CWRU dataset. The predicted labels closely matched the true labels overall, with deviations occurring at only a few sample positions. This result indicates that the model accurately identified bearing conditions associated with different fault locations and defect severities.

**Figure 10:**
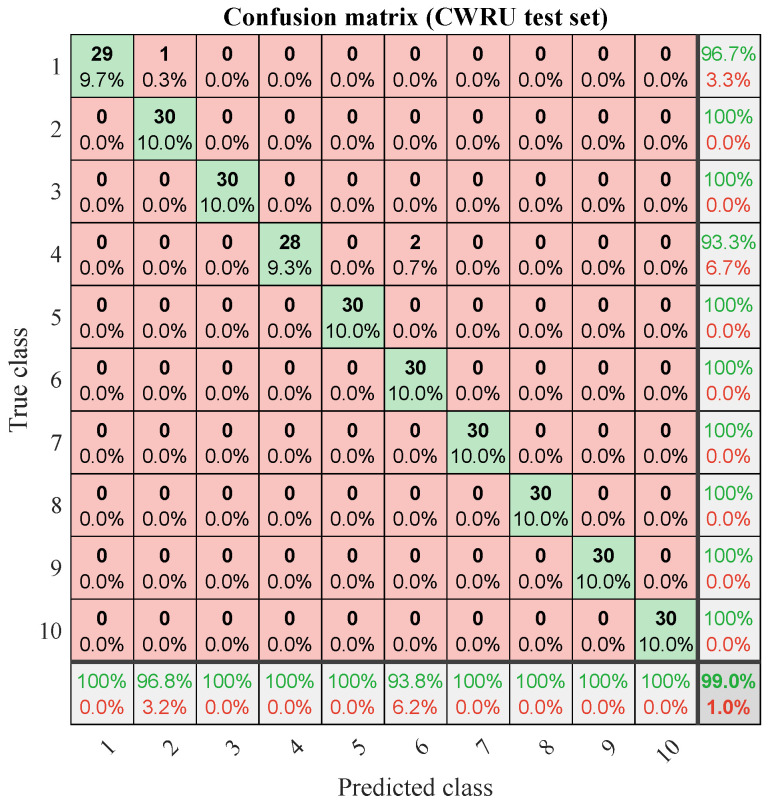
Confusion Matrix for the CWRU Dataset.

Figure 10 presents the corresponding confusion matrix. Except for Classes 1 and 4, all samples from the remaining eight classes were classified correctly, yielding a class-specific accuracy of 100%. For Class 1, 29 samples were correctly identified, whereas one sample was misclassified as Class 2, resulting in an accuracy of 96.7%. For Class 4, 28 samples were correctly identified, whereas two samples were misclassified as Class 6, resulting in an accuracy of 93.3%. Among the 300 test samples, 297 were correctly classified, yielding an overall accuracy of 99.0% and a misclassification rate of only 1.0%. The training time of MGFR-ViT on the CWRU dataset was 67.150 s, and the average inference time per sample was 12.902 ms. Sequential inference for all 300 test samples required approximately 3.871 s.

Overall, the proposed method achieved strong classification performance on the CWRU bearing dataset and accurately distinguished the normal condition and bearing faults with different locations and defect severities. Although slight confusion remained between a few similar fault conditions, interclass interference was limited overall.

### 4.3. Ablation Study

**Figure 11:**
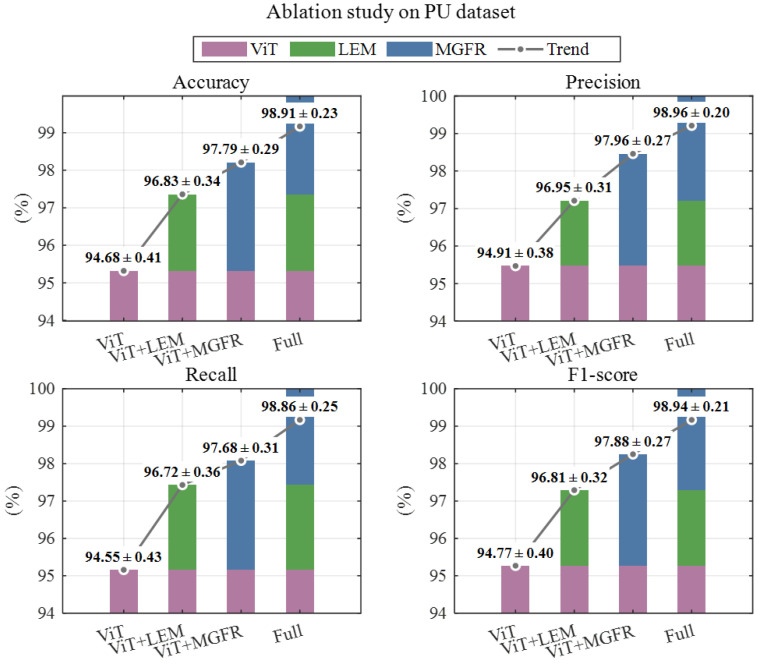
Ablation Experiment Results on the PU Dataset.

To evaluate the effectiveness of the key components in MGFR-ViT, ablation studies were conducted on the PU dataset using ViT as the baseline. LEM and MGFR were progressively incorporated to quantify their respective contributions to diagnostic performance. To reduce the influence of random data partitioning and model initialization, each configuration was independently evaluated over 10 runs. The corresponding means and standard deviations of the evaluation metrics are reported in Table 4 and Figure 11.

As shown in Table 4, the baseline ViT achieved an Accuracy, Precision, Recall, and F1-score of 94.68% ± 0.41%, 94.91% ± 0.38%, 94.55% ± 0.43%, and 94.77% ± 0.40%, respectively. These results indicate that ViT effectively modeled global dependencies in the vibration signals but remained limited in representing local impacts and fine-grained fault characteristics.

After LEM was introduced, Accuracy increased to 96.83% ± 0.34%, while Recall and F1-score reached 96.72% ± 0.36% and 96.81% ± 0.32%, respectively. This improvement demonstrates that LEM enhanced the extraction of local anomalous responses and weak fault signatures. When MGFR was introduced independently, Accuracy further increased to 97.79% ± 0.29%, and Precision reached 97.96% ± 0.27%. These results confirm that multi-scale feature extraction, channel interaction, and gated refinement effectively improved feature discriminability while suppressing redundant information.

When LEM and MGFR were incorporated simultaneously, the complete model achieved the best overall performance, with an Accuracy, Precision, Recall, and F1-score of 98.91% ± 0.23%, 98.96% ± 0.20%, 98.86% ± 0.25%, and 98.94% ± 0.21%, respectively. Compared with the baseline ViT, these four metrics increased by 4.23, 4.05, 4.31, and 4.17 percentage points, respectively. In addition, the standard deviations of all metrics were substantially reduced, indicating that the complete model not only achieved higher diagnostic accuracy but also exhibited greater stability under different random data partitions and model initializations.

Figure 11 visually illustrates the variation in the four-evaluation metrics under different module configurations. All metrics improved progressively as LEM and MGFR were introduced. LEM primarily strengthened the representation of local fault responses, whereas MGFR further improved multi-scale feature fusion and discriminative feature learning. The complete model achieved the best results across all four metrics, confirming the complementarity and synergistic effect of the two modules.

Overall, the quantitative results in Table 4 and the trends shown in Figure 11 demonstrate that both LEM and MGFR independently improved model performance. Their joint integration simultaneously strengthened global dependency modeling, local anomaly perception, and multi-scale feature refinement, thereby enabling more accurate and stable fault diagnosis of rotating machinery.

### 4.4. Comparative Experiment

To evaluate the effectiveness of MGFR-ViT in fault diagnosis, several representative deep learning models were selected for comparison. These models cover basic convolutional networks, classical convolutional Neural Network-based fault diagnosis models, deep convolutional architectures, standard self-attention models, and ViT-based models, enabling a comprehensive assessment of the proposed method in terms of fault feature extraction and classification performance. To ensure a fair comparison, all models were trained using the same data partitioning strategy, number of training epochs, optimizer, and evaluation metrics. Each model was independently evaluated over 10 runs, and the final results are reported as the mean ± standard deviation.

(1) CNN: A classical convolutional neural network was adopted, consisting of multiple convolutional layers, batch normalization layers, activation functions, and pooling layers. The convolution kernel size was set to 3 × 3.
(2) WDCNN: A wide-kernel convolutional neural network was used. A large convolution kernel was applied in the first convolutional layer to enhance the extraction of impact components and low-frequency fault features from raw vibration signals.
(3) ResNet18: A residual network structure was adopted. Residual connections were introduced to alleviate gradient vanishing and feature degradation during deep network training, thereby improving deep feature representation capability.
(4) VggNet16: A classical deep convolutional neural network was used. It was constructed by stacking multiple consecutive convolutional layers with small kernels and pooling layers, so that discriminative fault features could be progressively extracted from shallow to deep levels.
(5) Transformer: A standard Transformer encoder structure was adopted. Multi-head self-attention was used to model global dependencies among features, thereby enhancing the ability of the model to capture long-range correlation information.
(6) Swin Transformer: Window-based self-attention and shifted-window mechanisms were employed to achieve local window modeling and cross-window information interaction. This model was used to extract hierarchical fault features from two-dimensional vibration representations.

As shown in Table 5, substantial performance differences were observed among the models on the PU dataset. CNN and WDCNN achieved mean Accuracy values of 72.74% ± 0.62% and 77.95% ± 0.55%, respectively. As the network depth increased, the Accuracy values of ResNet18 and VGGNet16 improved to 82.66% ± 0.49% and 87.82% ± 0.43%, respectively. Transformer and Swin-Transformer further increased the Accuracy to 91.61% ± 0.38% and 94.72% ± 0.35%. In comparison, MGFR-ViT achieved the highest Accuracy of 98.91% ± 0.23%, outperforming Swin-Transformer by 4.19 percentage points. Its Precision, Recall, and F1-score were also close to 99%, demonstrating high diagnostic accuracy and stability.

Table 6 shows a similar trend on the UC dataset. MGFR-ViT achieved a mean Accuracy of 99.28% ± 0.18%, exceeding that of Swin-Transformer by 3.66 percentage points. Its Precision, Recall, and F1-score reached 99.34% ± 0.17%, 99.23% ± 0.19%, and 99.30% ± 0.16%, respectively, indicating strong recognition capability in gear fault diagnosis.

As shown in Table 7, CNN and WDCNN achieved mean Accuracy values of 70.86% ± 0.73% and 76.91% ± 0.65%, respectively, whereas ResNet18 and VGGNet16 reached 81.74% ± 0.57% and 86.93% ± 0.48%. The Accuracy values of Transformer and Swin-Transformer further increased to 90.81% ± 0.42% and 94.37% ± 0.34%, respectively. MGFR-ViT again achieved the best performance, with an Accuracy of 98.14% ± 0.27%, surpassing Swin-Transformer by 3.77 percentage points. In addition, its Precision, Recall, and F1-score all exceeded 98%, demonstrating strong feature extraction and classification capabilities across different bearing fault conditions and signal distributions.

Overall, Table 5, Table 6 and Table 7 show that MGFR-ViT consistently outperformed all comparison models across the PU, UC, and CWRU datasets, while also exhibiting relatively small standard deviations. These results demonstrate that local enhancement and multi-scale gated feature refinement effectively compensate for the limitations of conventional CNNs and Transformers in capturing local fault responses, integrating multi-scale information, and suppressing redundant features. Consequently, MGFR-ViT maintained high diagnostic accuracy and stability across different experimental platforms, mechanical components, and data distributions, further confirming its generalization capability.

## 5. Conclusions

An MGFR-ViT model was proposed for vibration-based fault diagnosis of rotating machinery. By embedding an LEM into the feed-forward network of ViT, LE-ViT was constructed and further integrated with MGFR, enabling the joint modeling of global dependencies, local fault responses, and multi-scale discriminative information.

Repeated experiments on the PU and CWRU bearing datasets and the UC gear dataset showed that MGFR-ViT outperformed several representative models in terms of Accuracy, Precision, Recall, and F1-score while maintaining favorable result stability. Ablation studies further confirmed the effectiveness and complementarity of LE-ViT and MGFR in local feature enhancement and multi-scale feature refinement.

MGFR-ViT demonstrates strong diagnostic performance and practical potential for vibration-based fault diagnosis of rotating machinery. Based on the real-time performance tests and considering the model size and inference overhead, the recommended deployment configuration comprises a quad-core processor, 8 GB of RAM, and a CUDA-compatible GPU with at least 4 GB of memory. Future work will focus on lightweight deployment, adaptive diagnosis across varying operating conditions, and online fault identification in real-world industrial environments.

## Figures and Tables

**Table 1:** Selected Rolling Bearing Fault Types in the PU Dataset.

Label	Fault Type	Fault Location	Fault Description	Number of Samples
1	K001	No fault	Normal condition	100
2	K002	No fault	Normal condition	100
3	K003	No fault	Normal condition	100
4	K004	No fault	Normal condition	100
5	KA01	Outer race	Artificial damage	100
6	KA03	Outer race	Artificial damage	100
7	KI01	Inner race	Artificial damage	100
8	KI03	Inner race	Artificial damage	100
9	KA04	Outer race	Real damage	100
10	KA15	Outer race	Real damage	100
11	KI04	Inner race	Real damage	100
12	KI14	Inner race	Real damage	100

**Table 2:** Selected Gear Fault Categories in the UC Dataset.

Label	Fault Type	Fault Location	Number of Samples
1	Healthy	None	104
2	Missing tooth	Tooth	104
3	Root crack	Tooth root	104
4	Spalling	Tooth surface	104
5	Chipping tip 5a	Tooth tip	104
6	Chipping tip 4a	Tooth tip	104
7	Chipping tip 3a	Tooth tip	104
8	Chipping tip 2a	Tooth tip	104
9	Chipping tip 1a	Tooth tip	104

**Table 3:** Selected Rolling Bearing Fault Types in the CWRU Dataset.

Label	Fault Type	Fault Location	Number of Samples
1	Normal	None	100
2	B-07	Rolling element	100
3	B-07	Rolling element	100
4	B-07	Rolling element	100
5	IR-07	Inner race	100
6	IR-07	Inner race	100
7	IR-07	Inner race	100
8	OR-07	Outer race	100
9	OR-07	Outer race	100
10	OR-07	Outer race	100

**Table 4:** Ablation Experiment Results of Different Modules on the PU Dataset.

ViT	LEM	MGFR	Accuracy (%)	Precision (%)	Recall (%)	F1-Score (%)
√			94.68 ± 0.41	94.91 ± 0.38	94.55 ± 0.43	94.77 ± 0.40
√	√		96.83 ± 0.34	96.95 ± 0.31	96.72 ± 0.36	96.81 ± 0.32
√		√	97.79 ± 0.29	97.96 ± 0.27	97.68 ± 0.31	97.88 ± 0.27
√	√	√	98.91 ± 0.23	98.96 ± 0.20	98.86 ± 0.25	98.94 ± 0.21

**Table 5:** Performance comparison of different methods on the PU dataset.

Model	Accuracy (%)	Precision (%)	Recall (%)	F1-Score (%)
CNN	72.74 ± 0.62	73.31 ± 0.58	72.38 ± 0.66	72.81 ± 0.61
WDCNN	77.95 ± 0.55	78.38 ± 0.51	77.62 ± 0.59	77.98 ± 0.54
ResNet18	82.66 ± 0.49	83.08 ± 0.45	82.31 ± 0.52	82.67 ± 0.48
VggNet16	87.82 ± 0.43	88.21 ± 0.40	87.38 ± 0.47	87.78 ± 0.44
Transformer	91.61 ± 0.38	91.18 ± 0.41	90.43 ± 0.44	90.77 ± 0.40
Swin Transformer	94.72 ± 0.35	95.04 ± 0.29	94.31 ± 0.34	94.65 ± 0.31
Proposed	98.91 ± 0.23	98.96 ± 0.20	98.86 ± 0.25	98.94 ± 0.21

**Table 6:** Performance comparison of different methods on the UC dataset.

Model	Accuracy (%)	Precision (%)	Recall (%)	F1-Score (%)
CNN	73.89 ± 0.64	74.31 ± 0.61	73.55 ± 0.67	73.91 ± 0.63
WDCNN	79.03 ± 0.56	79.46 ± 0.53	78.70 ± 0.60	79.06 ± 0.56
ResNet18	83.82 ± 0.48	84.27 ± 0.46	83.44 ± 0.51	83.84 ± 0.48
VggNet16	88.73 ± 0.42	89.16 ± 0.39	88.35 ± 0.45	88.73 ± 0.42
Transformer	91.89 ± 0.37	92.31 ± 0.35	91.49 ± 0.40	91.88 ± 0.37
Swin Transformer	95.62 ± 0.29	95.97 ± 0.27	95.27 ± 0.31	95.61 ± 0.29
Proposed	99.28 ± 0.18	99.34 ± 0.17	99.23 ± 0.19	99.30 ± 0.16

**Table 7:** Performance comparison of different methods on the CWRU dataset.

Model	Accuracy (%)	Precision (%)	Recall (%)	F1-Score (%)
CNN	70.86 ± 0.73	71.42 ± 0.69	70.31 ± 0.77	70.79 ± 0.81
WDCNN	76.91 ± 0.65	77.38 ± 0.61	76.44 ± 0.69	76.86 ± 0.64
ResNet18	81.74 ± 0.57	82.19 ± 0.53	81.26 ± 0.60	81.68 ± 0.56
VggNet16	86.93 ± 0.48	87.46 ± 0.45	86.39 ± 0.52	86.88 ± 0.49
Transformer	90.81 ± 0.42	91.16 ± 0.39	90.32 ± 0.46	90.72 ± 0.46
Swin Transformer	94.37 ± 0.34	94.76 ± 0.31	93.91 ± 0.37	94.29 ± 0.34
Proposed	98.14 ± 0.27	98.28 ± 0.24	98.28 ± 0.22	98.13 ± 0.26

## Data Availability

The raw bearing data used in this study are publicly available from the Paderborn University Bearing Data Center at https://mb.uni-paderborn.de/kat/forschung/bearing-datacenter/data-sets-and-download (accessed on 2 June 2026). The raw gear data are publicly available through Figshare at https://doi.org/10.6084/m9.figshare.28934972. The processed datasets and related experimental data supporting the findings of this study are available from the corresponding author upon reasonable request. The raw CWRU bearing data are publicly available from the Case Western Reserve University Bearing Data Center at https://engineering.case.edu/bearingdatacenter/download-data-file (accessed on 2 June 2026).
